# Effectiveness of calcium hydroxide compared to hydraulic calcium silicate cements for direct pulp capping in managing deep caries in vital permanent teeth: A systematic review and meta‐analysis

**DOI:** 10.1111/iej.14256

**Published:** 2025-05-20

**Authors:** Sascha R. Herbst, Vinay Pitchika, Chantal S. Herbst, Esra Kosan, Falk Schwendicke

**Affiliations:** ^1^ Department of Conservative Dentistry and Periodontology University Hospital, Ludwig‐Maximilian‐University of Munich Munich Germany; ^2^ Department for Periodontology, Oral Medicine and Oral Surgery Charité‐Universitätsmedizin Berlin Berlin Germany

**Keywords:** calcium hydroxide, direct pulp capping, hydraulic calcium silicate cements, meta‐analysis, systematic review

## Abstract

**Background:**

Direct pulp capping (DPC) is the least minimal approach for maintaining pulp vitality after pulp exposure. Besides calcium hydroxide (CaOH), hydraulic calcium silicate cements (HCSC) have increasingly been used for DPC.

**Objectives:**

During the S3 level guideline development for material choice in DPC, we conducted a systematic review and meta‐analysis of existing literature comparing CaOH and HCSC for DPC on permanent teeth. We aimed (1) to assess clinical and radiographic outcomes and (2) patient‐reported outcomes of DPC.

**Method:**

Three databases from 1 January 1990 to 19 February 2025 (MEDLINE via PubMed, EMBASE and Cochrane Database of Systematic Reviews). Prospective comparative clinical studies comparing CaOH to HCSC for DPC in permanent teeth with healthy or reversibly inflamed pulps were included. Studies on primary teeth or teeth indicative of irreversible pulpitis, teeth with unclear diagnoses or pulp exposure of non‐cariogenic origin were excluded. The risk of bias and certainty of evidence were evaluated using the GRADE approach. Using the effect sizes and standard errors for every study, pairwise meta‐analysis was performed comparing CaOH and different subgroups of HCSC. Success was defined as the absence of any clinical symptoms (e.g. pain, swelling) and any radiographical signs of an apical lesion. Patient‐reported outcomes were additionally sought after.

**Results:**

Five randomized‐controlled trials including 552 teeth with an overall moderate certainty of evidence were included. HCSC showed a significantly higher probability of success compared to CaOH (Odds Ratio (OR): 2.68, 95% confidence interval [1.7, 4.22], *I* ^2^ = 0%). The differences between various HCSC materials were minimal. Meta‐regression indicated that neither follow‐up nor risk of bias significantly influenced treatment outcomes, and the funnel plot did not reveal evidence of publication bias.

**Conclusion:**

HCSC showed significantly higher probability for clinical and radiographic success than CaOH. This finding comes with moderate certainty. The impact of material choice on postoperative pain remains unclear. Future clinical studies should include patient‐reported outcomes.

## INTRODUCTION

Endodontic treatment comprises maintaining or achieving a healthy pulp status and preventing or treating apical periodontitis (Duncan et al., 2019). Generally, a vital pulp is associated with higher chances of tooth survival than root canal treated teeth (Caplan et al., 2005). In addition, restorations placed on vital teeth showed higher longevity than endodontically treated teeth (Lempel et al., 2019; Lucarotti et al., 2014). Maintaining pulp vitality has been found cost‐effective by a number of studies in different healthcare systems and under different perspectives (Brodén et al., 2016; Emara et al., 2020).

The most common reason for an endodontic treatment is the management of caries and its sequelae (Scavo et al., 2011). A number of position statements and guidelines for managing caries lesions and maintaining pulp vitality have been published (Banerjee et al., 2017; Schwendicke et al., 2016, 2021), including a position statement of the European Society of Endodontology (ESE) (Duncan et al., 2019). According to these statements, the depth of the caries lesion and the clinical status of the pulp determine the subsequent treatment strategy. In teeth with vital pulps and no signs of irreversible pulpitis, but extremely deep caries lesions (radiographically penetrating the entire thickness of the dentin into the pulp), pulp exposure is often unavoidable and should be managed by direct pulp capping (DPC) or pulpotomy (partial and complete). In teeth with similar pulp conditions, but not extremely deep caries lesions (radiographically reaching the inner third or quarter of dentin, but with a radiographically detectable zone of dentin between lesion and pulp), selective carious tissue removal is recommended to avoid pulp exposure (Duncan et al., 2019). If pulp exposure nevertheless occurs, again DPC or pulpotomy are options.

In both DPC and pulpotomy, the pulp wound is covered with a biologically active material after achieving haemostasis to preserve pulp vitality, stimulate reparative dentin formation and prevent bacterial recontamination. For more than ninety years, calcium hydroxide (CaOH) has been used for this purpose (Dammaschke, 2008), and its efficacy was evaluated in clinical studies with up to 35 years follow‐up (Ricucci et al., 2023). However, CaOH showed some adverse effects on the pulp tissue, including local inflammation and incomplete reparative dentin formation. Furthermore, CaOH shows poor mechanical stability and is solvable to oral fluids, which can lead to degradation over time. To encounter the aforementioned disadvantages of CaOH, new material classes were developed, like hydraulic calcium silicate‐based cements (HCSC). HCSC are set through a hydration reaction after mixing with water‐containing liquids and offer high mechanical stability after several days. Besides that, HCSC release ions during the setting reaction like Ca, OH and Si, which have antibacterial as well as positive pulpal effects leading to reparative dentin formation with fewer tunnel defects and higher thicknesses compared to CaOH (Prati & Gandolfi, 2015). Reported disadvantages of HCSC were tooth discoloration potential, difficult handling characteristics and higher costs, while these disadvantages differ between different HCSC materials (Eskandari et al., 2022). The most frequently investigated HCSC materials for DPC are mineral trioxide aggregate (MTA, varying manufacturers) and Biodentine (BD, Septodont, Saint‐Maur‐des‐Fossés, France) (Fasoulas et al., 2023).

Several systematic reviews and meta‐analyses assessed different DPC materials, while all of them have certain limitations, like including experimental pulp cappings on third molars (Nair et al., 2008), including only younger patients (Brodén et al., 2016), including variable pulp conditions and pooling DPC with pulpotomy (Paula et al., 2018) or not reflecting on patient‐reported outcomes (Cushley et al., 2020; Fasoulas et al., 2023). The latter include measures of symptoms or function as well as oral health‐related quality of life (OHRQoL, e.g. disease burden and psychological consequences) and are increasingly regarded as relevant for decision‐making in addition to clinical outcomes (like success of a therapy) (Black, 2013; Doğramacı & Rossi‐Fedele, 2023).

As part of developing the joint ESE/EFCD/ORCA S3 level clinical practice guidelines for managing deep caries, the present systematic review and meta‐analysis was conducted to compare CaOH with HCSC for DPC in permanent teeth for their impact on clinical and radiographic success, as well as patient‐reported outcomes.

## MATERIALS AND METHODS

### Protocol and registration

The systematic review follows the Preferred Reporting Items for Systematic Reviews and Meta‐analyses (PRISMA) guidelines (Page et al., 2021) for reporting and the PICO framework for structuring the following clinical question: ‘In patients with deep caries lesions in mature or immature permanent teeth associated with no symptoms or those of a reversible pulpitis (P), is DPC with CaOH (I) as effective as direct pulp capping with HCSCs (C), in terms of clinical and radiographic success as well as patient‐reported outcomes (O), with “clinical and radiographic success” as the most critical outcome?’

This systematic review was registered in PROSPERO (CRD42023446138).

### Information sources and search strategy

A comprehensive search of three databases from January 1st, 1990 to February 19th, 2025, including MEDLINE via PubMed, EMBASE and Cochrane Database of Systematic Reviews, was performed independently by two authors (SH and CH). The following terms were used (including synonyms and closely related words) for developing the MeSH search items (Table S1): caries, permanent teeth, pulp capping, calcium hydroxide and hydraulic calcium silicate cements. The terms were combined using Boolean operators ‘AND’ and ‘OR’. Reference lists of included studies were screened for eligible studies; only studies published in English were considered in the review. Studies were administered in a reference management software (Zotero 7.0.6, Corporation for Digital Scholarship, Vienna/Virginia, USA).

### Eligibility criteria

Inclusion criteria were developed using an expansion of PICO, PICOTS:

- P(atient population): Mature or immature permanent teeth showing deep or extremely deep caries with no symptoms or reversible pulpitis.
- I(ntervention): Direct pulp capping using calcium hydroxide.
- C(omparator): Direct pulp capping using hydraulic calcium silicate cement.
- O(utcome): Patient‐centred outcome (clinical and radiographic success) and patient‐reported outcome.
- T(ime): Clinical and radiographic success should have been measured at least 12 months after DPC, patient‐reported outcomes at least 7 days after DPC.
- S(tudy design): Prospective comparative clinical trials in primary or secondary care.

DPC needed to be performed on permanent teeth (both immature and mature) with deep caries lesions, accompanied by an explicitly stated pulpal diagnosis (asymptomatic pulp or reversible pulpitis) and the use of a dental dam. Clinical and radiographic success was assessed after at least twelve months of follow‐up and was defined as the absence of any clinical symptoms (pain, swelling, sensitivity to percussion) and a positive response to at least one sensibility test (cold or electric pulp testing). No radiographic sign of a periapical lesion indicative of apical periodontitis was considered a radiographic success. Our secondary outcome comprised patient‐reported outcomes, for example the occurrence of postoperative pain and the need for medication (analgesics) in a minimum observation period of 7 days.

### Exclusion criteria

Studies evaluating primary teeth or teeth indicative of irreversible pulpitis or unclear diagnoses were excluded. Pulp exposure of non‐cariogenic origin, unrestored pulp exposures and teeth treated without dental dam were excluded.

### Selection of the studies

**FIGURE 1:**
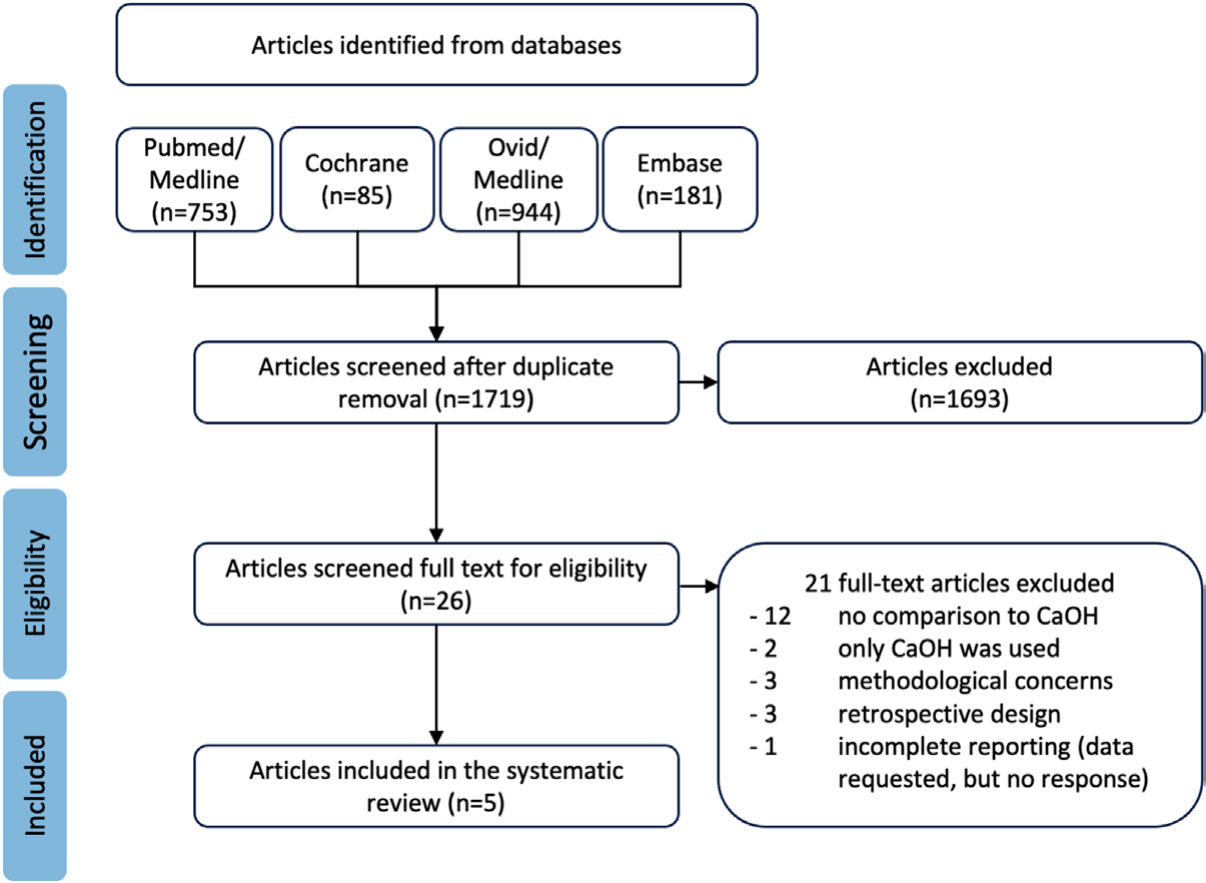
PRISMA flow diagram.

Two authors (SH, CH) independently screened titles and abstracts; the PRISMA flow diagram (Figure 1) depicts the selection process. Full texts were assessed for all studies potentially meeting the inclusion criteria or when the titles or abstracts in the screening phase were insufficient to apply the inclusion criteria. In cases of disagreement, a third author (FS) was consulted.

### Data extraction

Two authors (SH, CH) independently extracted the following information from each included study using a standardized form in an Excel spreadsheet (Microsoft Excel, Microsoft Office 2021, Redmond, USA): number of treated teeth and patients, interventions, sample size of each group, drop‐out rate, age of participants, pulpal diagnosis, use of dental dam, time allowed for haemostasis, coronal restoration, follow‐up protocol, assessed teeth, teeth with complications (with reasons) and patient‐reported outcomes.

### Quality assessment

Two independent authors (SH, CH) assessed the risk of bias using the RoB‐2 tool of the Cochrane Collaboration in the following domains: Randomization process, deviations from the intended interventions, missing outcome data, as well as selection of the reported results (Sterne et al., 2019). After evaluating each study separately for risk of bias and quality of evidence, the overall body of evidence was evaluated using the Grading of Recommendations Assessment, Development and Evaluation (GRADE) system (Balshem et al., 2011), under the following domains: risk of bias, inconsistency, indirectness, imprecision and publication bias.

### Meta‐analysis

This review compared different DPC materials (CaOH, HCSC, specifically MTA and BD) for their clinical and radiographical success. One study (Peskersoy et al., 2021) did not disclose the number of patients assessed or treated; therefore, the presence of clustering in that study could not be evaluated. The number of events in a certain instance (Brizuela et al., 2017) was zero. In order to avoid the exclusion of these studies, zero‐cell correction was performed. This involved adding 1 to both the number of teeth assessed and the number of events in all studies where such instances occurred in a group (Higgins et al., 2023a). Furthermore, a couple of included studies (Brizuela et al., 2017; Peskersoy et al., 2021) adopted a multi‐arm design, comprising three distinct groups. In such cases, the control group's (CaOH) total number of assessed teeth and the corresponding number of events were divided by two to facilitate comparison with the other two groups (Higgins et al., 2023b). This approach ensured a balanced assessment across all arms of the study. Pairwise meta‐analysis yielding Odds Ratios (OR) and their corresponding 95% Confidence Intervals (CI) was performed using the ‘metafor’ package in R, with additional stratifications by DPC material and follow‐up periods (12 and 36 months). Heterogeneity was measured using the *I* ^2^ statistic, and publication bias was tested using Harbord's test statistic (Harbord et al., 2009) using the ‘metabias’ in R. Harbord's test is particularly suitable for binary outcomes with a small number of studies, offering a more robust assessment of publication bias than Egger's test, which was initially planned. Moreover, although the number of included studies was limited, we conducted stratified meta‐analyses based on follow‐up duration. To further explore potential sources of heterogeneity, we performed meta‐regression analysis to assess whether follow‐up time and/or risk of bias had an impact on the pooled effect estimates (Higgins et al., 2023a).

## RESULTS

### Study selection

The initial search yielded 1963 entries (PubMed/Medline = 753, Cochrane = 85, Ovid/Medline = 944, Embase = 181). After removing duplicates automatically by the reference management software, abstracts and titles of 1719 studies were manually screened (Figure 1). Subsequently, 26 studies were identified for full‐text analysis and checked for eligibility. 21 studies did not meet the inclusion criteria (Table 1), and five studies were included in the present systematic review (Brizuela et al., 2017; Kundzina et al., 2017; Peskersoy et al., 2021; Suhag et al., 2019; Yavuz et al., 2025); all included studies were randomized‐controlled clinical trials. An additional hand search did not yield any additional relevant studies.

**TABLE 1:** Excluded studies after full‐text screening with reason.

Author and year	Reason for exclusion
Awawdeh 2018, Bogen 2008, Farsi 2006, Katge 2017, Kusumvalli 2019, Parinyaprom 2018, Holiel 2021, Shobana 2022, Soumya 2021, Yazdanfar 2020	No comparison was made to CaOH
Hilton 2013, Sabrah 2017, Parameswaran 2023, Alhuisany 2025	No standardized clinical protocol or insufficient reporting or no dental dam
Cho 2013, Mente 2010, Mente 2014	Retrospective study
Bjørndal 2010, Moritz 1998	Only CaOH was used

### Study characteristics

Table 2 presents a summary of all included studies in this systematic review. The sample sizes varied from 23 to 105 teeth per group and comprised mandibular permanent molars (Suhag et al., 2019), mandibular and maxillary permanent molars (Brizuela et al., 2017; Kundzina et al., 2017; Peskersoy et al., 2021) or premolars and molars (Yavuz et al., 2025). Patients' age ranged from 7 to 69 years and follow‐up time ranged from 12 months (Brizuela et al., 2017; Suhag et al., 2019; Yavuz et al., 2025) to 36 months (Kundzina et al., 2017; Peskersoy et al., 2021). No study reported any conflicts of interest, nor was any study funded by the manufacturer under investigation.

**TABLE 2:** Summary of all included studies.

Reference study design	Participants	Drop‐out rate and follow‐up	Intervention I	Intervention II	Control	Outcome(s)	Results
Suhag et al. (2019), RCT	64 teeth (CaOH 32, MTA 32); age 15‐40a; mean: 21.8 ± 5.9 Inclusion: occlusal deep caries penetrating more than half the thickness or more into dentin in mature permanent mandibular molars, closed apex on periapical, reversible pulpitis, pulpal vitality was confirmed by a positive response to cold and electrical tests and by the presence of bleeding after pulp exposure. Exclusion: primary teeth, lack of pulp exposure after excavation, teeth with irreversible pulpitis (spontaneous pain) or pulp necrosis, presence of periapical lesions as assessed by radiographic examination, periodontal disease, a cracked tooth, internal or external resorption, calcified canals, non‐restorable teeth, pulp bleeding that could not be controlled within 10 min using 2.5% sodium hypochlorite (NaOCl), immunocompromised or pregnant patients, patients with any systemic disorder and a positive history of antibiotic and analgesic use within the week before the treatment	CaOH: 3/32, 9.4% MTA: 5/32, 15.6% Follow‐up: 12 months	non‐selective caries removal; disinfection of the exposed pulp with 2.5% NaOCl using a syringe and a cotton pellet; haemostasis: cotton pellet was left for 10 min; removal of excess NaOCl with sterile saline (NaCl); MTA (White ProRoot; Dentsply, Tulsa, USA) was mixed (1:3 water/powder ratio) and applied to the exposure site with a sterile carrier; cotton pellet soaked in normal saline was placed over MTA; cavity was sealed temporarily with intermediate restorative material (IRM, Dentsply Tulsa Dental); after 24 h: setting of MTA was evaluated, layer of resin‐modified glass ionomer cement liner (Fusion iseal, Prevest DenPro Limited, Jammu, India) was placed followed by direct composite restoration.		non‐selective caries removal; disinfection of the exposed pulp with 2.5% NaOCl using a syringe and a cotton pellet; haemostasis: cotton pellet was left for 10 min; removal of excess NaOCl with sterile saline (NaCl); CaOH (Prevest DenPro) powder mixed with saline and applied over the exposure site followed by a layer of RMGIC liner and direct composite restoration (Te‐Econom Plus; Ivoclar Vivadent AG, Schaan, Germany).	primary outcome: clinical and radiographic success secondary outcome: postoperative pain up to 7 days	Absolute effects: CaOH: 9 failures MTA: 2 failures Comparison: Success rate: CaOH 69.0%; MTA 92.6%; *p* = 0.026 Significantly lower pain scores were reported in the MTA group (6.3 ± 9.5) compared with the CaOH group (18.5 ± 20.8) after 18 h
Kundzina et al. (2017), RCT	70 teeth (CaOH 37, MTA 33); age 18‐55a Inclusion: permanent molar with a proximal carious lesion (primary or secondary caries); no history of pain or the presence of pain indicating a reversible pulpitis; positive response to a cold test or to electric pulp, bitewing radiograph showing a carious lesion in at least the inner 1/3 of the dentine; periapical radiograph showing closed apex and normal periapex (with no radiolucency or widening of the periodontal ligament space); attachment loss not exceeding 4 mm; non‐contributory medical history (including pregnancy); and no use of medication (no antibiotics during the previous month). Only one pulp cap was to be included per subject Exclusion: lack of pulpal exposure after complete removal of the caries and failure to control the bleeding in exposed pulp within 10 min	CaOH: 3/37, 8.1% MTA: 2/33, 6.1% Follow‐up: 36 months	non‐selective caries excavation; haemostasis within 1–10 min with NaOH‐soaked cotton pellet (0.5%); mixed white MTA (Dentsply, Tulsa Dental, Tulsa, OK, USA) was applied to the pulpal exposure (2 mm layer) and a moistened cotton pellet was placed directly over the material; temporary GIC filling (Fuji IX glass ionomer cement; GC Corp, Tokyo, Japan); after 7 d composite (not specified) restoration (parts of the temporary filling were left to protect the MTA)	NA	non‐selective caries excavation; haemostasis within 1–10 min with NaOH‐soaked cotton pellet (0.5%); thin layer of CaOH (Dycal, Dentsply DeTrey GmbH, Konstanz, Germany) was applied to the pulpal exposure and was left to set; temporary GIC filling (Fuji IX glass ionomer cement; GC Corp, Tokyo, Japan); after 7 d composite (not specified) restoration (parts of the temporary filling were left to protect the CaOH)	primary outcome: clinical and radiographic success secondary outcome: Postoperative pain after 1 week	Absolute effects: Absolute effects not clearly stated Comparison: cumulative estimate rate: CaOH 52%; MTA 85%; *p* = 0.006 no significant associations between the material and postoperative pain (*p* > 0.05) Statistics: effect size: 0.25 number needed to treat (NTT) = 3
Brizuela et al. (2017), RCT	169 teeth (CaOH 53, MTA 56, BD 60); age 7 ‐16a (mean 11.3a) Inclusion: less than 2 mm of carious exposure in a permanent molar, with complete or incomplete radicular growth, and with pulpal testing that was compatible with normal pulp or reversible pulpitis Exclusion: patients with systemic and/or neurologic pathology, teeth with radiologic signs of internal resorption or pulpal calcifications, no restorable teeth and uncontrollable pulpal bleeding	CaOH: 31/53 (58.5%) MTA: 34/56 (60.7%) BD: 35/60 (58.3%) Follow‐up: 12 months	Non‐selective caries removal using a caries detector dye (Sable Seek; Ultradent Products Inc., South Jordan, USA) to standardize all the treatments; haemostasis up to 10 min by applying pressure over the exposed pulp with cotton pellets soaked with sterile saline; white MTA (ProRoot MTA; Dentsply Maillefer, USA) was applied to the pulp exposure; glass ionomer liner (Vitrebond; 3 M ESPE, St Paul, MN) was placed; final restoration with composite (Filtek Z350 XT Universal Restorative; 3 M ESPE)	Non‐selective caries removal using a caries detector dye (Sable Seek) to standardize all the treatments; haemostasis up to 10 min by applying pressure over the exposed pulp with cotton pellets soaked with sterile saline; BD (Septodont, Saint‐Maur‐des‐Fossés, France) was applied to the pulp exposure; glass ionomer liner (Vitrebond; 3 M ESPE, St Paul, MN) was placed; final restoration with composite (Filtek Z350 XT Universal Restorative; 3 M ESPE)	Non‐selective caries removal using a caries detector dye (Sable Seek) to standardize all the treatments; haemostasis up to 10 min by applying pressure over the exposed pulp with cotton pellets soaked with sterile saline; CaOH (Hertz Pharmaceutical, Santiago, Chile) was applied to the pulp exposure; glass ionomer liner (Vitrebond; 3 M ESPE, St Paul, MN) was placed; final restoration with composite (Filtek Z350 XT Universal Restorative; 3 M ESPE)	Clinical and radiographic success	Absolute effects: CaOH: 3 failures MTA: 3 failures BD: 0 failures Comparison: Success rate: CaOH 86.4%; MTA 86.4%; BD 100% Statistics: P > 0.05
Peskersoy et al. (2021), RCT	315 teeth (CaOH 105, MTA 105, BD 105); age 18‐42a Inclusion: class II profound caries with no signs of extraoral/intraoral symptoms or pulpal exposure while radiographic findings revealed caries in close proximity to the pulp; negative to percussion and palpation tests; mobility within normal limits; pulp vitality tests using vitalometer (Parkell, Edgewood, NY, USA) and cold spray test showed positive response; pulp diagnosis: reversible pulpitis Exclusion: history of systemic diseases; teeth showing clinical and radiographic evidence of pulp degeneration such as history of spontaneous or nocturnal pain, tenderness to percussion or palpation, necrosis of the pulp (negative to vitality tests), swelling or fistulous tract, pathologic mobility due to aggressive periodontitis, periodontal ligament space widening (PDL), internal root resorption, external root resorption, furcal radiolucency/inter‐radicular bone destruction and/or periapical bone destruction	Not reported explicitly: CaOH: 0/105 (0%) MTA: 0/105 (0%) BD: 0/105 (0%) Follow‐up: 36 months	Non‐selective caries removal; haemostasis up to 5 min by applying pressure over the exposed pulp with sterile cotton pellets soaked with sterile saline; MTA (BioMTA+, Cerkamed, Stalowa Wola, Poland) was applied according to the manufacturers' instructions; universal self‐etching adhesive system (Beauti Bond, Shofu Corp, Tokyo, Japan) and nanohybrid composite resin (NCR) (Beautifil II, Shofu Corp)	Non‐selective caries removal; haemostasis up to 5 min by applying pressure over the exposed pulp with sterile cotton pellets soaked with sterile saline; BD (Septodont) was applied according to the manufacturers' instructions; universal self‐etching adhesive system (Beauti Bond, Shofu Corp, Tokyo, Japan) and nanohybrid composite resin (NCR)	Non‐selective caries removal; haemostasis up to 5 min by applying pressure over the exposed pulp with sterile cotton pellets soaked with sterile saline; CaOH (Dycal, Dentsply‐Sirona) was applied according to the manufacturers' instructions; universal self‐etching adhesive system (Beauti Bond, Shofu Corp, Tokyo, Japan) and nanohybrid composite resin (NCR)	Clinical and radiographic success	Absolute effects: CaOH: 32 failures MTA: 16 failures BD: 22 failures Comparison: Success rate: CaOH 69.4%; MTA 86.3%; BD 79.4% Statistics: *p* > 0.05
Yavuz et al. (2025)	49 teeth (CaOH 23, BD 26); age 17‐69a Inclusion: Vital teeth with no history of spontaneous or long‐lasting pain (thermal/chemical stimuli), no percussion sensitivity, pathological mobility, oedema, fistula or colour change; radiographically healthy lamina dura	CaOH: 1/23 (4.3%) BD: 1/26 (3.8%) Follow‐up 12 months	Non‐selective caries removal, haemostasis with sterile cotton and moderate pressure for 5 min; BD was applied according to the manufacturers' instructions; universal adhesive (G‐Premio Bond; GC, Tokyo, Japan) and Filtek Universal Composite (3 M)	Non‐selective caries removal, haemostasis with sterile cotton and moderate pressure for 5 min; CaOH (Dycal) was applied according to the manufacturers' instructions; universal adhesive (G‐Premio Bond; GC, Tokyo, Japan) and Filtek Universal Composite (3 M)		Clinical and radiographic success	Absolute effects: CaOH: 7 failures BD: 1 failure Comparison: Success rate: CaOH 68.2%; BD 96.0% Statistics: *p* > 0.05

Three studies allowed up to 10 min for haemostasis (Brizuela et al., 2017; Kundzina et al., 2017; Suhag et al., 2019), whereas two studies allowed for 5 min (Peskersoy et al., 2021; Yavuz et al., 2025). Two studies compared MTA with CaOH (Kundzina et al., 2017; Suhag et al., 2019), one study compared BD with CaOH (Yavuz et al., 2025) and two studies compared MTA and BD to CaOH (Brizuela et al., 2017; Peskersoy et al., 2021). In four studies, the definitive coronal restoration was placed either immediately after the pulp capping (Brizuela et al., 2017; Peskersoy et al., 2021; Yavuz et al., 2025) or in a second visit after 2–7 days (Kundzina et al., 2017) for all treatment groups. In one study, the CaOH group received an immediate coronal restoration whereby the MTA group received a temporary restoration for 24 h (Suhag et al., 2019).

All included studies evaluated the clinical and radiographic success of DPC: Three studies found statistically significant differences between HCSC and CaOH (Kundzina et al., 2017; Suhag et al., 2019; Yavuz et al., 2025) and two studies found no statistically significant differences between CaOH and HCSC (Brizuela et al., 2017; Peskersoy et al., 2021). Two of the included studies evaluated postoperative pain as a patient‐reported outcome.

### Quality assessment

**FIGURE 2:**
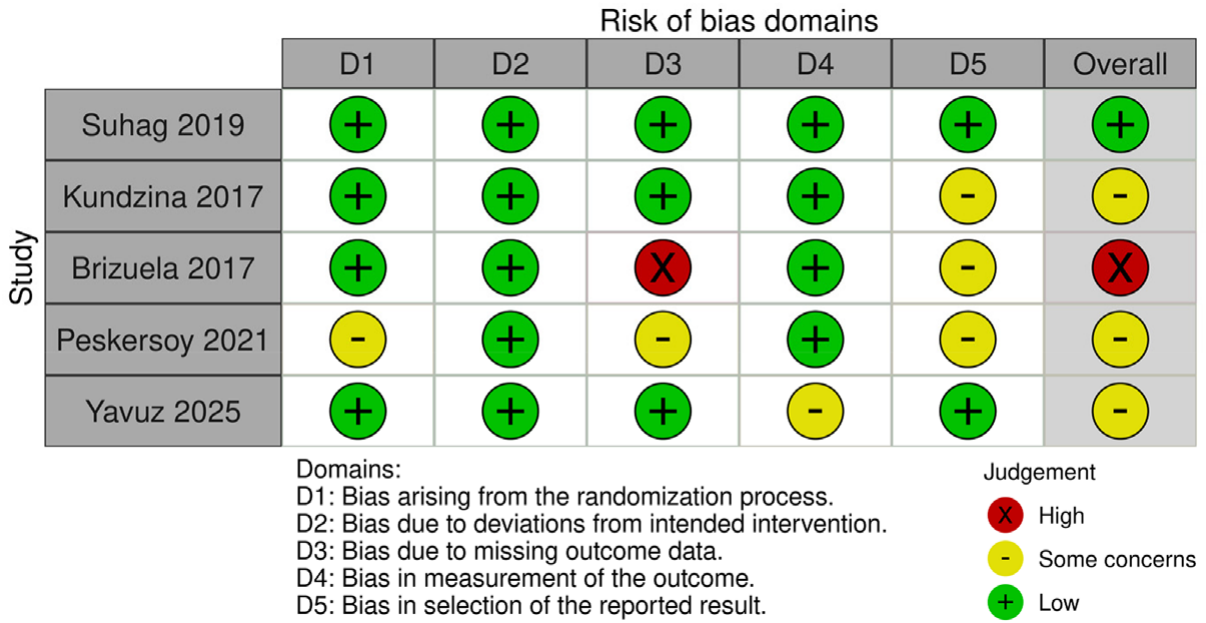
Risk of bias assessment of individual studies.

Five studies were analysed for risk of bias. One study was considered to have a high quality and a low risk of bias; four studies showed some (severe) concerns; these were: (1) calculated sample size could not be reached (Kundzina et al., 2017); (2) loss to follow‐up not reported (Peskersoy et al., 2021); (3) drop‐out rate 59% (Brizuela et al., 2017); (4) small sample size (treated teeth: 23 CaOH/26 BD). Table 3 and Figure 2 outline the details of the risk of bias assessment (McGuinness & Higgins, 2020).

**TABLE 3:** Results of risk of bias analysis using RoB 2.

Study (author/year)	Study design	Randomization process	Risk of bias domains					Quality rating	Remarks
			Deviations from the intended interventions	Missing outcome data	Measurement of the outcome	Selection of the reported results	Other sources of bias*		
Suhag et al. (2019)	RCT	Low	Low	Low	Low	Low	No	High	No remarks
Kundzina et al. (2017)	RCT	Low	Low	Low	Low	Some concerns	Yes	Some concerns	Downgrade: calculated sample size could not be reached; only reporting of the cumulative survival rate and no absolute numbers of failures
Brizuela et al. (2017)	RCT	Low	Low	High	Low	Some concerns	Yes	Low	Downgrade: high drop‐out rate: 59%
Peskersoy et al. (2021)	RCT	Some concerns	Low	Some concerns	Low	Some concerns	Yes	Some concerns	Downgrade: no loss to follow‐up reported
Yavuz et al. (2025)	RCT	Low	Low	Low	Low	Low	Yes	Some concerns	Small sample size

### Clinical and radiographic success

**FIGURE 3:**
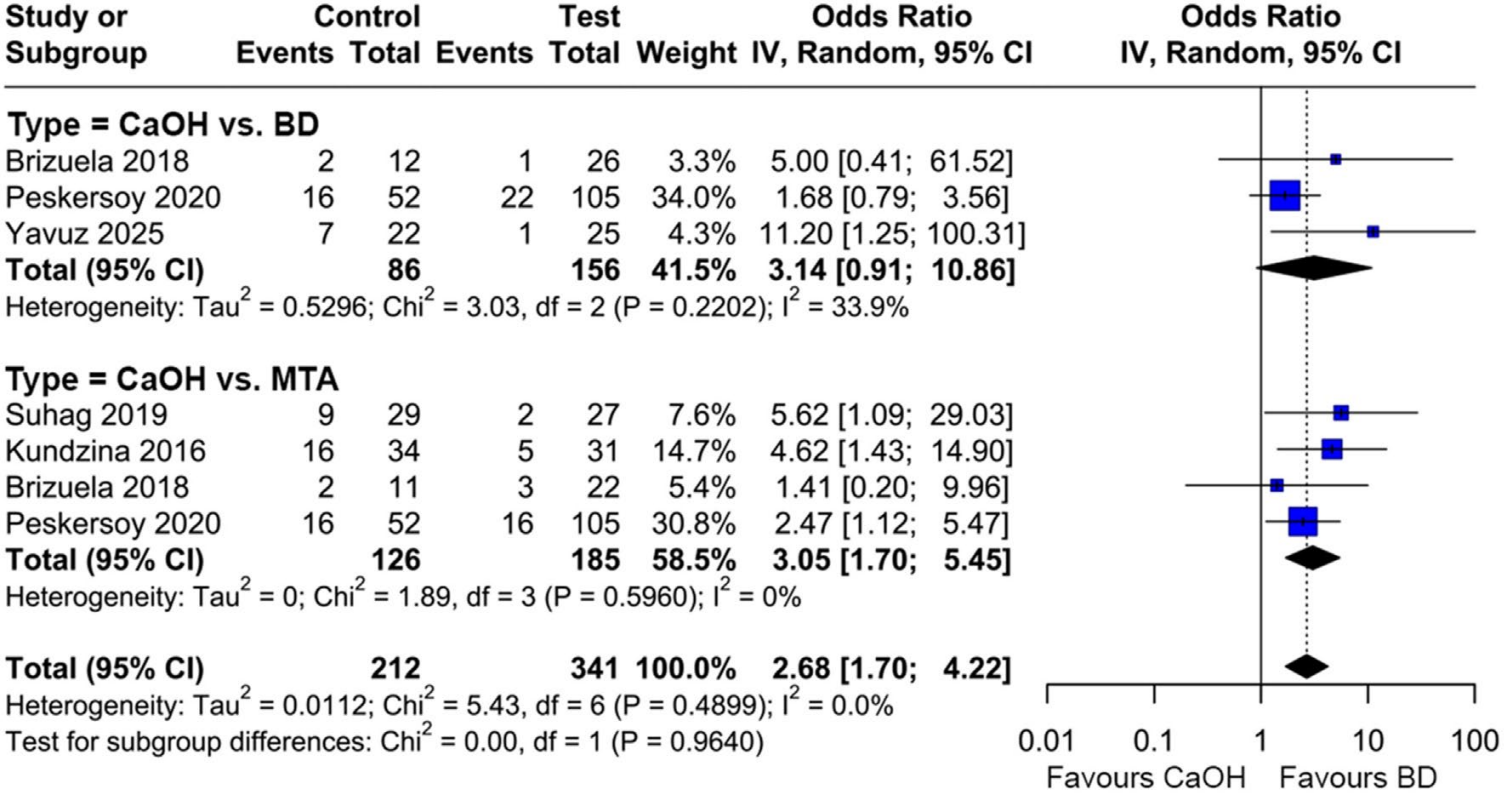
Forest plot comparing HCSC with CaOH for radiographic and clinical success of DPC, stratified by the pairwise comparison; CaOH: Calcium hydroxide, BD: Biodentine, MTA: Mineral trioxide aggregate.

**FIGURE 4:**
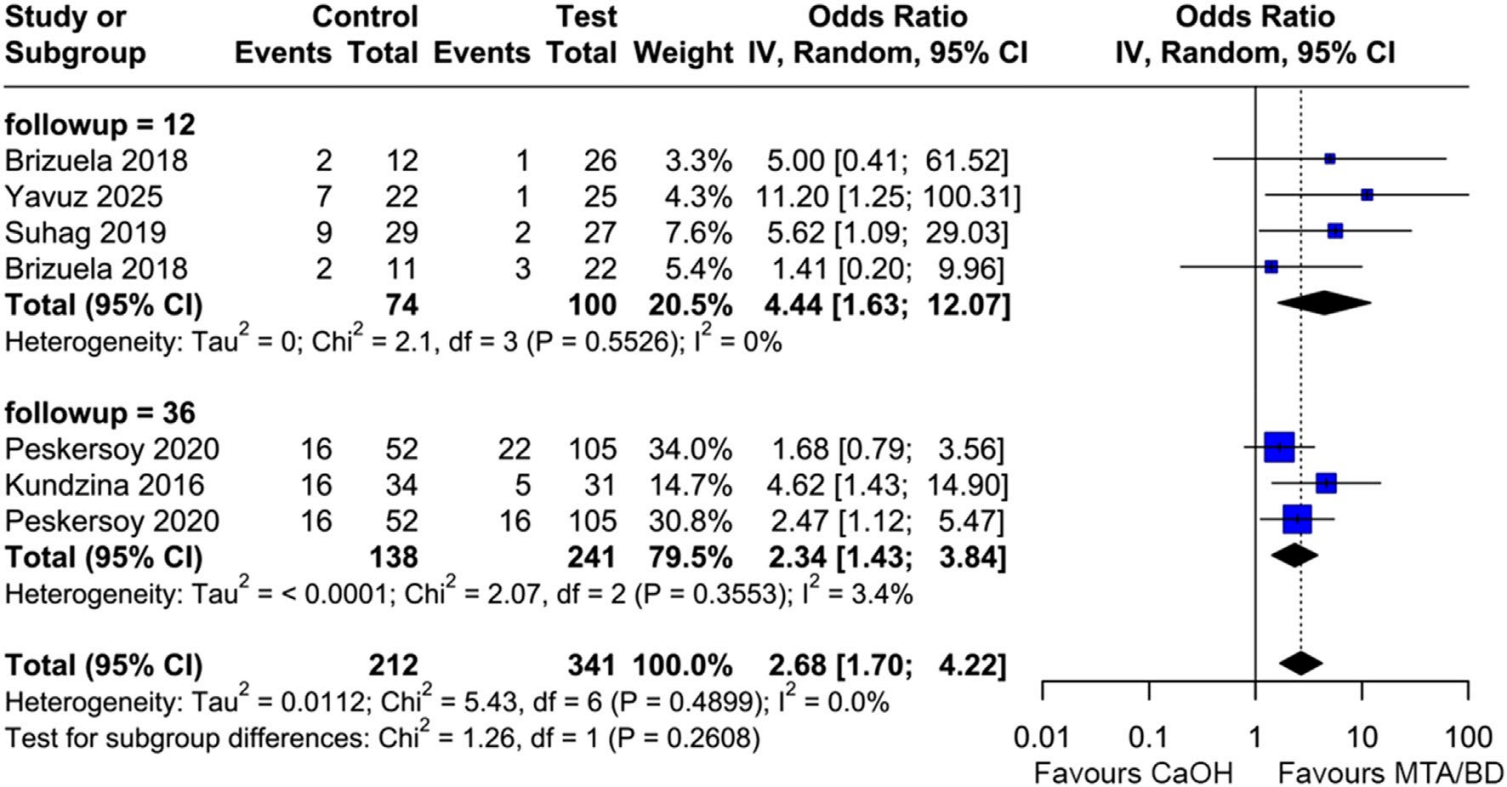
Forest plot for comparing HCSC with CaOH for radiographic and clinical success of DPCs, stratified by the follow‐up time in months; CaOH: Calcium hydroxide, BD: Biodentine, MTA: Mineral trioxide aggregate.

MTA and BD were pooled as HCSC for the overall comparison against CaOH. HCSC showed a statistically significant higher probability for clinical and radiographic success (OR: 2.68, 95% CI [1.7, 4.22], *I* ^2^ = 0%) than CaOH (Figure 3). When comparing BD against CaOH in three studies including 242 patients (CaOH: 86, BD: 156) (Brizuela et al., 2017; Peskersoy et al., 2021; Yavuz et al., 2025), no statistically significant difference between both materials could be observed (OR 3.14, 95% CI [0.91, 10.86], *I* ^2^ = 33.9%) (Figure 3). The comparison between MTA and CaOH was conducted on 311 patients in four studies (CaOH: 126, MTA: 185 patients) (Brizuela et al., 2017; Kundzina et al., 2017; Peskersoy et al., 2021; Suhag et al., 2019) and MTA performed significantly better than CaOH (OR 3.05, 95% CI [1.70, 5.45], *I* ^2^ = 0%). Subgroup analysis stratified for the follow‐up time (12 months) (Brizuela et al., 2017; Suhag et al., 2019; Yavuz et al., 2025) and 36 months (Kundzina et al., 2017; Peskersoy et al., 2021) revealed statistically significant differences in the meta‐analyses (12 months with 174 patients: OR 4.44, 95% CI [1.63, 12.07], *I* ^2^ = 0%; 36 months with 379 patients: OR 2.34, 95% CI [1.43, 3.84], *I* ^2^ = 3.4%) (Figure 4).

### Postoperative pain

Two studies assessed postoperative pain. Due to heterogeneity within the associated methodology, we did not perform meta‐analysis for the outcome postoperative pain. In the first study, a visual‐analogue scale (100 mm) was used to assess postoperative pain after 6, 12, 18 h and daily within 7 days; additionally, analgesic intake was noted (ibuprofen 400 mg every 6‐8 h) (Suhag et al., 2019). A significant reduction in pain was observed after 6 h in both groups compared to pre‐treatment pain score (CaOH: 35.06 ± 24.75, MTA: 35.16 ± 26.57, *p* > 0.05); after 18 h, significantly higher pain scores were reported in the CaOH group compared to the MTA group (CaOH: 18.5 ± 20.8 mm, MTA: 6.3 ± 9.5 mm, *p* < 0.05), but there was no statistically significant difference in mean analgesic consumption (CaOH: 1.13 ± 2.63, MTA: 0.19 ± 0.78, *p* > 0.05). In the second study, patients were asked whether they had any pain at present or experienced any pain during the first 3 days after treatment (Kundzina et al., 2017). There was no significant difference in the reported prevalence or experience of pain between groups (CaOH: 21.6%, MTA: 30.3%, p > 0.05).

### Grading of evidence

A critical appraisal of the included studies was performed for grading the overall body of evidence:

- Risk of bias: 10.1% of all assessed treatments were derived from high‐quality studies with low risk of bias and 12.5% from low‐quality studies with high risk of bias; so 77.4% of all data were provided by studies with moderate quality. The overall risk of bias was considered to be moderate.
- Imprecision: Comparing CaOH with HCSC, the confidence interval covered treatment effects only in one direction (favourable for HCSC). Hence, a downgrade was not justified.
- Inconsistency: Despite having a limited number of included studies resulting in a potentially biased *I* ^2^ (Von Hippel, 2015), we found overlapping confidence intervals for each effect size and the test for heterogeneity indicated low variability (*p* > 0.05 using Harbord's test statistic). Hence, we did not downgrade.
- Indirectness: All studies included patients, interventions and outcomes of interest; no indirectness was detected.
- Publication bias: Due to the small number of studies per subgroup (k = 2 to 4), a formal test for small‐study effects, such as Harbord's test, could not be performed. However, when Harbord's test was performed on the pooled dataset (9 pairwise comparisons from 5 studies), the test did not indicate significant funnel plot asymmetry (t = 1.49, df = 7, *p* = 0.18), suggesting no statistically significant evidence of small‐study effects; in other words, no publication bias. However, given the small number of included studies, these findings should be interpreted with caution. No study reported an industry sponsor.
- Heterogeneity: Due to the limited number of studies in each follow‐up, we performed a meta‐regression to assess the influence of follow‐up time and risk of bias on the pooled estimate. It was found that neither follow‐up time (β = −1.824 [−4.33; 0.69], *p* = 0.154) nor risk of bias (medium: β = 0.69 [−2.40; 3.78], *p* = 0.663; high: β = −0.66 [−3.08; 1.75], *p* = 0.590) had a significant impact on effect estimates. The resulting model resulted in a Q‐statistic of 3.05 (*p* = 0.384), indicating that neither follow‐up nor risk of bias significantly influenced the effect estimates.

Rating the body of evidence: Due to serious concerns about the risk of bias, the body of evidence was rated with a moderate certainty of evidence.

## DISCUSSION

DPC is considered the most minimal approach for managing the exposed pulp. The question of material choice has been under discussion for many decades (Shovelton et al., 1971), and several systematic reviews and meta‐analyses aimed to summarize the current evidence with various focuses, as outlined earlier. Our systematic review and meta‐analysis aimed to investigate the radiographical and clinical success as well as patient‐reported outcomes of CaOH and HCSC for DPC in prospective comparative clinical studies. We found that HCSC showed a significantly higher probability of clinical and radiographical success rate compared to CaOH with a moderate certainty of evidence. For patient‐reported outcomes, uncertainty remains. Patient reported lower or similar postoperative pain when MTA versus CaOH was used. Our findings need some in‐depth discussion.

Our primary outcome estimate aligns with recent systematic reviews and meta‐analyses (Cushley et al., 2020; Fasoulas et al., 2023). Notably, subgroup analyses found MTA showing a significantly higher probability of success compared with CaOH than BD. However, it should be noted that data on BD was relatively sparse, involved longer follow‐up, and also the risk of bias differed between groups. One study with low quality reported a 100% success rate for BD and equal success rates for CaOH and MTA (86.4%) after 12 months but came with a drop‐out rate of 59%. While this study contributed to meta‐analysis, its robustness needs to be challenged. Overall, we conclude that the underlying data are insufficient to identify significant differences between different HCSC, in line with a recent systematic review (Cushley et al., 2020). Notably, the uncertainty around the comparison between different HCSC has also been found for pulpotomy (Ather et al., 2022; Silva et al., 2023). Further research should therefore not focus solely on comparing HCSC against CaOH, but rather on comparing different types of HCSC against each other. For instance, tooth discoloration may influence patient‐reported outcomes, and various HCSC exhibit varying staining effects (Palma et al., 2020). In this research, further outcomes like applicability or cost‐effectiveness should be reflected.

One of these additional, relevant outcomes is patient reports. In the present review, only two studies reported on such outcomes. One study indicated some benefit of MTA over CaOH after some months, while another did not demonstrate this. However, these findings only represent short time follow‐ups. Another prospective clinical study (not included here) assessed third molars (sound or with incipient caries, scheduled for extraction) capped either with CaOH or MTA and evaluated pain after 7 days. This study found no statistically significant difference between both materials (Iwamoto et al., 2006). Moreover, a recent study indicates that postoperative pain is also dependent on the agent used for pulp lavage; using sterile saline was associated with a higher proportion of painful failures compared to 1% NaOCl (sterile saline: 66%; 1% NaOCL 33%) (Ballal et al., 2023). Accordingly, postoperative pain seems not only to depend on the material used for pulp capping, and any differences found seem of limited robustness and certainty. The paucity of patient‐reported outcomes in endodontics has been criticized before (Azarpazhooh, Sgro, et al., 2022). Future clinical studies should integrate patient‐centred and patient‐reported outcomes to comprehensively evaluate the suitability of different treatments (Doğramacı & Rossi‐Fedele, 2023). Moreover, these outcomes should be collected using comparable (ideally standardized) outcome measures (El Karim et al., 2022).

Factors associated with the success of DPC are a matter of ongoing discussion. Recently, long‐term follow‐up studies (retrospective and prospective) investigating factors associated with the success of DPC were published; one study reported outcomes from up to four years follow‐up (Ballal et al., 2023) and another study up to 35 years (Ricucci et al., 2023). They report conflicting results: Ricucci et al. (2023) observed a success rate of 89% for DPC with CaOH after 35 years and advocated for performing DPC, whereas Ballal et al. 2023 found an estimated pulp survival of 55% (95% CI [30%, 100%]) after 1500 days when lavaging with 1% NaOCl and capping with MTA. Both studies emphasized the need for aseptic conditions during the management of the exposed pulp and also highlighted the relevance of operators' experience. Ricucci et al., 2023 reported a sophisticated clinical protocol, which might be difficult to implement for generalists in most health care settings (e.g. professional tooth cleaning prior to initiating the treatment, changing and using sterile armamentarium before entering the pulp tissue, use of high magnification and adaptation of the treatment protocol depending on the pulp exposure size). Additionally, the disinfection protocol had also a significant impact in the study of Ballal et al. 2023; by using sterile saline (without antimicrobial effect) for pulp lavage instead of 1% NaOCl (with an antimicrobial effect), the estimated pulp survival dropped to 7% (95% CI [1%, 40%]. In both studies, specialists performed the treatments. When DPC is performed by undergraduate students, the success rate of DPC of cariously exposed pulps was considerably low at 33% after three years (Al‐Hiyasat et al., 2006). Overall, these findings highlight the complex nature of DPC, in which the material choice is just one of multiple confounding factors including aseptic conditions, pulp lavage and operator experience. Given the high success rate of DPC only achieved with a sophisticated clinical protocol (Ricucci et al., 2023) and considering the limited success rate observed in most other studies (Al‐Hiyasat et al., 2006; Bjørndal et al., 2010; Hilton et al., 2013), the routine use of DPC in a primary care setting without further training can be seen critically.

There are a number of methodological aspects to discuss. First, the different included studies contributed differently to our analyses. One study contributed 57% of all included teeth (315 of 552 teeth) (Peskersoy et al., 2021), which had an impact on the critical appraisal of the body of evidence. The varying sample sizes of the included studies raised some concerns regarding the sample size calculation, too. Two studies reported no sample size calculation at all (Brizuela et al., 2017; Peskersoy et al., 2021), one study reported a sample size calculation but was not able to include the required number of patients (Kundzina et al., 2017) and two studies estimated the sample size and met it (Suhag et al., 2019; Yavuz et al., 2025). None of the included studies performed a post hoc power analysis. Limited or unclear power is a common phenomenon in medicine (and endodontics); the majority of Cochrane reviews has been found to include (and synthesize) underpowered studies (Turner et al., 2013). However, the total number of included patients in this study exceeded with 552 teeth the proposed threshold of 398 teeth (according to the post hoc sample size calculation of Çalışkan & Güneri, 2017), and the optimal information size could be achieved (Schünemann et al., 2013). Second, we assessed heterogeneity and found it to be limited, but given the small number of included studies the estimated *I* ^2^ may be erroneous (Von Hippel, 2015). Hence, we can only limitedly infer towards the true heterogeneity (and hence, to some degree, consistency and robustness) of our data. Third, our risk of bias analysis revealed study qualities ranging from low to high per study (Table 3), resulting in an overall of moderate certainty of evidence. That means that the true effect is likely to be close to the observed effect estimate but there is a possibility that it is substantially different due to the mentioned concerns (Balshem et al., 2011). In general, heterogenous quality among clinical studies is a common phenomenon in past endodontic research (Azarpazhooh, Cardoso, et al., 2022, p. 1); an important measure towards to increasing the quality in future clinical research is the establishment of a core outcome set (El Karim et al., 2022).

Our study comes with several strengths and limitations. First, clinical and patient‐reported outcomes were reflected upon, and given the strict inclusion criteria; a meta‐analysis could be conducted. Second, and as a limitation, only four studies were included, leading to a small sample size with inconclusive subgroup analyses. Besides that, only two studies reported postoperative pain as a patient‐reported outcome, each with a different methodology, so comparability and certainty are limited. Third, given this low number of studies, publication bias could not be assessed comprehensively for subgroup analyses. Fourth, although we had a limited number of studies, we stratified the pairwise comparisons by material type and follow‐up duration. While follow‐up time can influence failure rates – since failure may not occur at a constant rate over time – it is uncertain whether this time dependence would differ significantly between groups. To investigate this, we conducted a meta‐regression to assess whether follow‐up duration or risk of bias influenced the effect estimates. When follow‐up and RoB were included as moderators, no significant association with the effect size was observed (QM = 3.05, *p* = 0.384), indicating that neither follow‐up nor study‐level RoB significantly influenced the effect estimates. Additionally, the language restriction (considering only studies in English) can be perceived as a limitation, potentially contributing to the low number of included studies. However, it is worth noting that no eligible studies in different languages were identified during the screening process.

## CONCLUSION

Considering the strengths and limitations of this systematic review and meta‐analysis, we can conclude that HCSC have a higher probability of success in DPC than CaOH with moderate certainty of evidence; the underlying data are insufficient to identify significant differences between different HCSC. Postoperative pain was evaluated in two studies with different methodologies so that no meta‐analysis was conducted, and both studies reported contradicting results. Future research should focus on comparing different HCSC and should include patient‐reported outcomes as well as reflect on wider outcomes like applicability and cost‐effectiveness.

## AUTHOR CONTRIBUTIONS

All the authors have made relevant contributions to the manuscript. All the authors have read and approved the final version of the manuscript.

## FUNDING INFORMATION

This study received no external funding.

## CONFLICT OF INTEREST STATEMENT

The authors deny any conflicts of interest related to this study. Outside of this work, SR Herbst has been a consultant for Septodont (Saint‐Maur‐des‐Fossés, France).

## ETHICS STATEMENT

Not applicable. This article is secondary research involving published literature.

## Supporting information


**Table S1.** MeSH search items for literature search.

## Data Availability

The data that support the findings of this study are available from the corresponding author upon reasonable request.
