# Supplementary material for: Effectiveness of calcium hydroxide compared to hydraulic calcium silicate cements for direct pulp capping in managing deep caries in vital permanent teeth: A systematic review and meta‐analysis
Source: Int Endod J. 2025 May 20;58(8):1110–25. doi: 10.1111/iej.14256 (PMC12254524; doi:10.1111/iej.14256)
Supplement: Supplementary file 1 — Table S1. MeSH search items for literature search. [file IEJ-58-1110-s001.docx]

**Table S1** MeSH search items for literature search.

| **Nr.** | **PICOTS** | **Search terms** |
| --- | --- | --- |
| 1 | P | caries OR  carious lesion OR  tooth decay AND  direct pulp capping  MeSH:  Dental Caries AND  Dental Pulp Capping |
| 2 | P | Permanent Teeth OR  Secondary Dentition OR  Adult Teeth  MeSH:  Adult Dentition OR  Dentition, Adult OR  Dentition, Permanent OR  Secondary Dentition |
| 3 | I | Calcium hydroxide OR  Ca(OH)2  MeSH:  Calcium Hydroxide |
| 4 | C | Hydraulic calcium silicate cement OR  HCSC  MeSH:  Silicate Cement |
| 5 | S | Prospective comparative clinical trials in primary or secondary care. |
| 6 |  | #1 AND #2 AND #3 AND #4 |

(((caries OR carious lesion OR tooth decay) AND pulp capping) OR (Calcium hydroxide OR Ca(OH)2)) OR (Hydraulic calcium-silicate cements OR HCSCs)

Al-Hiyasat, A. S., Barrieshi-Nusair, K. M., & Al-Omari, M. A. (2006). The radiographic outcomes of direct pulp-capping procedures performed by dental students: A retrospective study. *Journal of the American Dental Association (1939)*, *137*(12), 1699–1705. https://doi.org/10.14219/jada.archive.2006.0116

Ather, A., Patel, B., Gelfond, J. A. L., & Ruparel, N. B. (2022). Outcome of pulpotomy in permanent teeth with irreversible pulpitis: A systematic review and meta-analysis. *Scientific Reports*, *12*(1), 19664. https://doi.org/10.1038/s41598-022-20918-w

Azarpazhooh, A., Cardoso, E., Sgro, A., Elbarbary, M., Laghapour Lighvan, N., Badewy, R., Malkhassian, G., Jafarzadeh, H., Bakhtiar, H., Khazaei, S., Oren, A., Gerbig, M., He, H., Kishen, A., & Shah, P. S. (2022). A Scoping Review of 4 Decades of Outcomes in Nonsurgical Root Canal Treatment, Nonsurgical Retreatment, and Apexification Studies—Part 1: Process and General Results. *Journal of Endodontics*, *48*(1), 15–28. https://doi.org/10.1016/j.joen.2021.09.018

Azarpazhooh, A., Sgro, A., Cardoso, E., Elbarbary, M., Laghapour Lighvan, N., Badewy, R., Malkhassian, G., Jafarzadeh, H., Bakhtiar, H., Khazaei, S., Oren, A., Gerbig, M., He, H., Kishen, A., & Shah, P. S. (2022). A Scoping Review of 4 Decades of Outcomes in Nonsurgical Root Canal Treatment, Nonsurgical Retreatment, and Apexification Studies—Part 2: Outcome Measures. *Journal of Endodontics*, *48*(1), 29–39. https://doi.org/10.1016/j.joen.2021.09.019

Ballal, N. V., Duncan, Henry. F., Wiedemeier, Daniel. B., Rai, N., Jalan, P., Bhat, V., Belle, V. S., & Zehnder, M. (2023). Four-Year Pulp Survival in a Randomized Trial on Direct Pulp Capping. *Journal of Endodontics*, S0099239923006532. https://doi.org/10.1016/j.joen.2023.10.008

Balshem, H., Helfand, M., Schünemann, H. J., Oxman, A. D., Kunz, R., Brozek, J., Vist, G. E., Falck-Ytter, Y., Meerpohl, J., & Norris, S. (2011). GRADE guidelines: 3. Rating the quality of evidence. *Journal of Clinical Epidemiology*, *64*(4), 401–406. https://doi.org/10.1016/j.jclinepi.2010.07.015

Banerjee, A., Frencken, J. E., Schwendicke, F., & Innes, N. P. T. (2017). Contemporary operative caries management: Consensus recommendations on minimally invasive caries removal. *British Dental Journal*, *223*(3), 215–222. https://doi.org/10.1038/sj.bdj.2017.672

Bjørndal, L., Reit, C., Bruun, G., Markvart, M., Kjaeldgaard, M., Näsman, P., Thordrup, M., Dige, I., Nyvad, B., Fransson, H., Lager, A., Ericson, D., Petersson, K., Olsson, J., Santimano, E. M., Wennström, A., Winkel, P., & Gluud, C. (2010). Treatment of deep caries lesions in adults: Randomized clinical trials comparing stepwise vs. direct complete excavation, and direct pulp capping vs. partial pulpotomy: Treatment of deep caries in adults. *European Journal of Oral Sciences*, *118*(3), 290–297. https://doi.org/10.1111/j.1600-0722.2010.00731.x

Black, N. (2013). Patient reported outcome measures could help transform healthcare. *BMJ*, *346*(jan28 1), f167–f167. https://doi.org/10.1136/bmj.f167

Brizuela, C., Ormeño, A., Cabrera, C., Cabezas, R., Silva, C. I., Ramírez, V., & Mercade, M. (2017). Direct Pulp Capping with Calcium Hydroxide, Mineral Trioxide Aggregate, and Biodentine in Permanent Young Teeth with Caries: A Randomized Clinical Trial. *Journal of Endodontics*, *43*(11), 1776–1780. https://doi.org/10.1016/j.joen.2017.06.031

Brodén, J., Heimdal, H., Josephsson, O., & Fransson, H. (2016). Direct pulp capping versus root canal treatment in young permanent vital teeth with pulp exposure due to caries. A systematic review. *American Journal of Dentistry*, *29*(4), 201–207.

Çalışkan, M. K., & Güneri, P. (2017). Prognostic factors in direct pulp capping with mineral trioxide aggregate or calcium hydroxide: 2- to 6-year follow-up. *Clinical Oral Investigations*, *21*(1), 357–367. https://doi.org/10.1007/s00784-016-1798-z

Caplan, D. J., Cai, J., Yin, G., & White, B. A. (2005). Root Canal Filled Versus Non-Root Canal Filled Teeth: A Retrospective Comparison of Survival Times. *Journal of Public Health Dentistry*, *65*(2), 90–96. https://doi.org/10.1111/j.1752-7325.2005.tb02792.x

Cushley, S., Duncan, H., Lappin, M., Chua, P., Elamin, A., Clarke, M., & El‐Karim, IA. (2020). Efficacy of direct pulp capping for management of cariously exposed pulps in permanent teeth: A systematic review and meta‐analysis. *International Endodontic Journal*. https://doi.org/10.1111/iej.13449

Dammaschke, T. (2008). The history of direct pulp capping. *Journal of the History of Dentistry*, *56*(1), 9–23.

Doğramacı, E. J., & Rossi-Fedele, G. (2023). Patient-related outcomes and Oral Health-Related Quality of Life in endodontics. *International Endodontic Journal*, *56*(S2), 169–187. https://doi.org/10.1111/iej.13830

Duncan, H. F., Galler, K. M., Tomson, P. L., Simon, S., El-Karim, I., Kundzina, R., Krastl, G., Dammaschke, T., Fransson, H., Markvart, M., Zehnder, M., & Bjørndal, L. (2019). European Society of Endodontology position statement: Management of deep caries and the exposed pulp. *International Endodontic Journal*, *52*(7), 923–934. https://doi.org/10.1111/iej.13080

El Karim, I., Duncan, H. F., Cushley, S., Nagendrababu, V., Kirkevang, L., Kruse, C., Chong, B. S., Shah, P. K., Lappin, M. J., McLister, C., Lundy, F. T., & Clarke, M. (2022). Establishing a Core Outcome Set for Endodontic Treatment modalities. *International Endodontic Journal*, *55*(7), 696–699. https://doi.org/10.1111/iej.13749

Emara, R., Krois, J., & Schwendicke, F. (2020). Maintaining pulpal vitality: Cost-effectiveness analysis on carious tissue removal and direct pulp capping. *Journal of Dentistry*, *96*, 103330. https://doi.org/10.1016/j.jdent.2020.103330

Eskandari, F., Razavian, A., Hamidi, R., Yousefi, K., & Borzou, S. (2022). An Updated Review on Properties and Indications of Calcium Silicate-Based Cements in Endodontic Therapy. *International Journal of Dentistry*, *2022*, 1–19. https://doi.org/10.1155/2022/6858088

Fasoulas, A., Keratiotis, G., Spineli, L., Pandis, N., De Bruyne, M. A. A., De Moor, R. J. G., & Meire, M. A. (2023). Comparative efficacy of materials used in patients undergoing pulpotomy or direct pulp capping in carious teeth: A systematic review and meta‐analysis. *Clinical and Experimental Dental Research*, cre2.767. https://doi.org/10.1002/cre2.767

Harbord, R. M., Harris, R. J., & Sterne, J. A. C. (2009). Updated Tests for Small-study Effects in Meta-analyses. *The Stata Journal: Promoting Communications on Statistics and Stata*, *9*(2), 197–210. https://doi.org/10.1177/1536867X0900900202

Hilton, T. J., Ferracane, J. L., Mancl, L., & Northwest Practice-based Research Collaborative in Evidence-based Dentistry (NWP). (2013). Comparison of CaOH with MTA for direct pulp capping: A PBRN randomized clinical trial. *Journal of Dental Research*, *92*(7 Suppl), 16S-22S. https://doi.org/10.1177/0022034513484336

Iwamoto, C. E., Adachi, E., Pameijer, C. H., Barnes, D., Romberg, E. E., & Jefferies, S. (2006). Clinical and histological evaluation of white ProRoot MTA in direct pulp capping. *American Journal of Dentistry*, *19*(2), 85–90.

Kundzina, R., Stangvaltaite, L., Eriksen, H. M., & Kerosuo, E. (2017). Capping carious exposures in adults: A randomized controlled trial investigating mineral trioxide aggregate versus calcium hydroxide. *International Endodontic Journal*, *50*(10), 924–932. https://doi.org/10.1111/iej.12719

Lempel, E., Lovász, B. V., Bihari, E., Krajczár, K., Jeges, S., Tóth, Á., & Szalma, J. (2019). Long-term clinical evaluation of direct resin composite restorations in vital vs. Endodontically treated posterior teeth—Retrospective study up to 13 years. *Dental Materials: Official Publication of the Academy of Dental Materials*, *35*(9), 1308–1318. https://doi.org/10.1016/j.dental.2019.06.002

Lucarotti, P. S. K., Lessani, M., Lumley, P. J., & Burke, F. J. T. (2014). Influence of root canal fillings on longevity of direct and indirect restorations placed within the General Dental Services in England and Wales. *British Dental Journal*, *216*(6), E14–E14. https://doi.org/10.1038/sj.bdj.2014.244

McGuinness, L. A., & Higgins, J. P. T. (2020). Risk-of-bias VISualization (robvis): An R package and Shiny web app for visualizing risk-of-bias assessments. *Research Synthesis Methods*, *n/a*(n/a). https://doi.org/10.1002/jrsm.1411

Nair, P. N. R., Duncan, H. F., Pitt Ford, T. R., & Luder, H. U. (2008). Histological, ultrastructural and quantitative investigations on the response of healthy human pulps to experimental capping with mineral trioxide aggregate: A randomized controlled trial. *International Endodontic Journal*, *41*(2), 128–150. https://doi.org/10.1111/j.1365-2591.2007.01329.x

Page, M. J., McKenzie, J. E., Bossuyt, P. M., Boutron, I., Hoffmann, T. C., Mulrow, C. D., Shamseer, L., Tetzlaff, J. M., Akl, E. A., Brennan, S. E., Chou, R., Glanville, J., Grimshaw, J. M., Hróbjartsson, A., Lalu, M. M., Li, T., Loder, E. W., Mayo-Wilson, E., McDonald, S., … Moher, D. (2021). The PRISMA 2020 statement: An updated guideline for reporting systematic reviews. *Journal of Clinical Epidemiology*, *134*, 178–189. https://doi.org/10.1016/j.jclinepi.2021.03.001

Palma, P. J., Marques, J. A., Santos, J., Falacho, R. I., Sequeira, D., Diogo, P., Caramelo, F., Ramos, J. C., & Santos, J. M. (2020). Tooth Discoloration after Regenerative Endodontic Procedures with Calcium Silicate-Based Cements—An Ex Vivo Study. *Applied Sciences*, *10*(17), 5793. https://doi.org/10.3390/app10175793

Paula, A. B., Laranjo, M., Marto, C.-M., Paulo, S., Abrantes, A. M., Casalta-Lopes, J., Marques-Ferreira, M., Botelho, M. F., & Carrilho, E. (2018). Direct Pulp Capping: What is the Most Effective Therapy?—Systematic Review and Meta-Analysis. *Journal of Evidence Based Dental Practice*, *18*(4), 298–314. https://doi.org/10.1016/j.jebdp.2018.02.002

Peskersoy, C., Lukarcanin, J., & Turkun, M. (2021). Efficacy of different calcium silicate materials as pulp-capping agents: Randomized clinical trial. *Journal of Dental Sciences*, *16*(2), 723–731. https://doi.org/10.1016/j.jds.2020.08.016

Prati, C., & Gandolfi, M. G. (2015). Calcium silicate bioactive cements: Biological perspectives and clinical applications. *Dental Materials: Official Publication of the Academy of Dental Materials*, *31*(4), 351–370. https://doi.org/10.1016/j.dental.2015.01.004

Ricucci, D., Rôças, I. N., Alves, F. R. F., Cabello, P. H., & Siqueira, J. F. (2023). Outcome of Direct Pulp Capping Using Calcium Hydroxide: A Long-term Retrospective Study. *Journal of Endodontics*, *49*(1), 45–54. https://doi.org/10.1016/j.joen.2022.11.005

Scavo, R., Martinez Lalis, R., Zmener, O., DiPietro, S., Grana, D., & Pameijer, C. H. (2011). Frequency and distribution of teeth requiring endodontic therapy in an Argentine population attending a specialty clinic in endodontics. *International Dental Journal*, *61*(5), 257–260. https://doi.org/10.1111/j.1875-595X.2011.00069.x

Schünemann, H., Brożek, J., Guyatt, G., Oxman, A., & editors. (2013). *GRADE handbook for grading quality of evidence and strength of recommendations. Updated October 2013. The GRADE Working Group, 2013.* Available from guidelinedevelopment.org/handbook.

Schwendicke, F., Frencken, J. E., Bjørndal, L., Maltz, M., Manton, D. J., Ricketts, D., Van Landuyt, K., Banerjee, A., Campus, G., Doméjean, S., Fontana, M., Leal, S., Lo, E., Machiulskiene, V., Schulte, A., Splieth, C., Zandona, A. F., & Innes, N. P. T. (2016). Managing Carious Lesions: Consensus Recommendations on Carious Tissue Removal. *Advances in Dental Research*, *28*(2), 58–67. https://doi.org/10.1177/0022034516639271

Schwendicke, F., Walsh, T., Lamont, T., Al-Yaseen, W., Bjørndal, L., Clarkson, J. E., Fontana, M., Gomez Rossi, J., Göstemeyer, G., Levey, C., Müller, A., Ricketts, D., Robertson, M., Santamaria, R. M., & Innes, N. P. (2021). Interventions for treating cavitated or dentine carious lesions. *The Cochrane Database of Systematic Reviews*, *7*(7), CD013039. https://doi.org/10.1002/14651858.CD013039.pub2

Shovelton, D. S., Friend, L. A., Kirk, E. E., & Rowe, A. H. (1971). The efficacy of pulp capping materials. A comparative trial. *British Dental Journal*, *130*(9), 385–391. https://doi.org/10.1038/sj.bdj.4802670

Silva, E. J. N. L., Pinto, K. P., Belladonna, F. G., Ferreira, C. M. A., Versiani, M. A., & De‐Deus, G. (2023). Success rate of permanent teeth pulpotomy using bioactive materials: A systematic review and meta‐analysis of randomized clinical trials. *International Endodontic Journal*, *56*(9), 1024–1041. https://doi.org/10.1111/iej.13939

Sterne, J. A. C., Savović, J., Page, M. J., Elbers, R. G., Blencowe, N. S., Boutron, I., Cates, C. J., Cheng, H.-Y., Corbett, M. S., Eldridge, S. M., Emberson, J. R., Hernán, M. A., Hopewell, S., Hróbjartsson, A., Junqueira, D. R., Jüni, P., Kirkham, J. J., Lasserson, T., Li, T., … Higgins, J. P. T. (2019). RoB 2: A revised tool for assessing risk of bias in randomised trials. *BMJ (Clinical Research Ed.)*, *366*, l4898. https://doi.org/10.1136/bmj.l4898

Suhag, K., Duhan, J., Tewari, S., & Sangwan, P. (2019). Success of Direct Pulp Capping Using Mineral Trioxide Aggregate and Calcium Hydroxide in Mature Permanent Molars with Pulps Exposed during Carious Tissue Removal: 1-year Follow-up. *Journal of Endodontics*, *45*(7), 840–847. https://doi.org/10.1016/j.joen.2019.02.025

Turner, R. M., Bird, S. M., & Higgins, J. P. T. (2013). The impact of study size on meta-analyses: Examination of underpowered studies in Cochrane reviews. *PloS One*, *8*(3), e59202. https://doi.org/10.1371/journal.pone.0059202

Von Hippel, P. T. (2015). The heterogeneity statistic I2 can be biased in small meta-analyses. *BMC Medical Research Methodology*, *15*(1), 35. https://doi.org/10.1186/s12874-015-0024-z
